# Evaluating the carbon balance estimate from an automated ground-level flux chamber system in artificial grass mesocosms

**DOI:** 10.1002/ece3.879

**Published:** 2013-11-11

**Authors:** Andreas Heinemeyer, Jemma Gornall, Robert Baxter, Brian Huntley, Phil Ineson

**Affiliations:** 1 Stockholm Environment Institute (SEI-York centre) at the Environment Department, University of YorkYork, YO10 5DD, UK; 2 Metoffice at the Hadley CentreFitzRoy Road, Exeter, EX1 3PB, UK; 3 School of Biological and Biomedical Sciences, Durham UniversityDurham, DH1 3LE, UK; 4 Department of Biology, University of YorkYork, YO10 5DD, UK

**Keywords:** Carbon balance, carbon fluxes, flux chamber, net ecosystem exchange, temperature response, validation

## Abstract

Measuring and modeling carbon (C) stock changes in terrestrial ecosystems are pivotal in addressing global C-cycling model uncertainties. Difficulties in detecting small short-term changes in relatively large C stocks require the development of robust sensitive flux measurement techniques. Net ecosystem exchange (NEE) ground-level chambers are increasingly used to assess C dynamics in low vegetation ecosystems but, to date, have lacked formal rigorous field validation against measured C stock changes. We developed and deployed an automated and multiplexed C-flux chamber system in grassland mesocosms in order rigorously to compare ecosystem total C budget obtained using hourly C-flux measurements *versus* destructive net C balance. The system combines transparent NEE and opaque respiration chambers enabling partitioning of photosynthetic and respiratory fluxes. The C-balance comparison showed good agreement between the two methods, but only after NEE fluxes were corrected for light reductions due to chamber presence. The dark chamber fluxes allowed assessing temperature sensitivity of ecosystem respiration (*R*_eco_) components (i.e., heterotrophic vs. autotrophic) at different growth stages. We propose that such automated flux chamber systems can provide an accurate C balance, also enabling pivotal partitioning of the different C-flux components (e.g., photosynthesis and respiration) suitable for model evaluation and developments.

## Introduction

Linking the terrestrial carbon (C) cycle to climate and to potential climatic change feedbacks has become a central focus of much C-cycle research across the globe. Both, actual field C-flux measurements and modeling of such data are pivotal to advancing our understanding of this fundamental biogeochemical cycle. Current model predictions suggest that the land surface will cease to be a net C sink by 2050, with large uncertainties in the biotic feedbacks (IPCC 2007); the largest uncertainty relates to the responses of soil organic carbon (SOC) stocks (Friedlingstein et al. 2006).

Soils represent the major reservoir of terrestrial organic C, but there are large uncertainties and difficulties in detecting soil C stock changes. Global SOC maps (e.g., ISLSCP II; ORNL DAAC, obtainable from http://daac.ornl.gov/) show particularly high SOC stocks in organic soils of short boreal (e.g., Northern Canada) and tropical (e.g., South-East Asia) vegetation and in peatlands, albeit with large uncertainties in the estimates (see Heinemeyer et al. 2010). Remarkably, total SOC stocks (particularly considering organic soils) are not being modeled accurately by existing global C-cycle models (Heinemeyer et al. 2010) nor are soil respiration fluxes (Trumbore 2006). Whereas about one-third of SOC occurs in forests, another third occurs in grasslands and savannas, and the remainder in wetlands, croplands and other mostly short vegetation biomes (Janzen 2004). Understanding the potential of, and uncertainties in, any terrestrial climatic change feedback from such biomes is important, as even small changes in these large SOC pools, due to climatic change or human activity, might have large impacts upon the global C cycle (Garten and Wullschleger 1999; Vance 2003). Thus, it becomes essential to assess accurately both C balance and SOC stock change in short vegetation biomes such as mires, fens, and grasslands that have large SOC stocks. However, as a result of the large background soil C content and the inherently high spatial and temporal heterogeneity (Niklaus et al. 2000), C stock changes (e.g., by sequential coring) are mostly below detection limits and large-scale measurements, for example from eddy covariance towers, do not capture the considerable spatial variability of such systems; a clear priority thus is making available small-scale chamber technology enabling detecting accurately any short-term changes in fluxes rather than stocks also capturing any spatial variability in ecosystem net C-flux balance.

Chamber-based methods are now widely used for short vegetation C-flux measurements, with examples including Hirota et al. (2010; alpine meadows), Stocker et al. (1997; grasslands), Huemmrich et al. (2010; tundra), Laine et al. (2007), Laine et al. (2009; peatlands). Importantly, such chamber systems potentially overcome the problems of detecting short-term changes in C stock inventories, being based on more precise detection of C-flux changes that should correspond to overall C-pool changes (Niklaus et al. 2000). Clearly, this chamber approach requires the use of accurate automated chamber equipment; although this is now available commercially (e.g., Li-Cor, USA or ADC, U.K.), it has never been strictly validated against C stock estimates based on mass balance. One concern is that flux measurement artifacts may result in calculation of misleading C stock changes.

Chamber fluxes further offer an important addition to eddy covariance and aircraft fluxes (see Myklebust et al. (2008) and Oechel et al. (1998)) as they address small-scale spatial variability. However, the level of accuracy of a flux approach is often assessed simply by comparison of different flux system approaches (e.g., Myklebust et al. 2008) or of automated versus manual sampling (e.g., Burrows et al. 2005) but, to date, never against absolute C stock changes. Although one manual chamber C-flux balance validation study has been reported for soil respiration (Nay and Bormann 2000), crucially, to the best of our knowledge, continuous NEE flux-based C-balance estimates have never been critically validated under field conditions against a mesocosm C stock balance inventory.

Although global C-cycle models have advanced over the past decade, not least because of computing power, some C-cycle process representations are still very uncertain. This is particularly true for SOC turnover and its environmental responses (Friedlingstein et al. 2006). Models require process-level uncertainties to be reduced further in order to improve predictions of future C sink *vs*. source relationships within ecosystems. Specifically, net primary productivity (NPP) modeling approaches are based on generalizations of GPP to NPP ratios, mostly empirically allocating assimilated C to either respiratory loss or biomass gain and thus determining turnover rates of C in ecosystems (Gifford 2003; Trumbore 2006). Moreover, in modeling ecosystems, environmental responses have mostly been treated uniformly (Williams et al. 2001; Shaver et al. 2007), ignoring responses of vegetation patches. Separating and explaining variability in measured chamber NEE flux components is key to overcoming these current measurement and model limitations.

The NEE flux is composed of two major components: C uptake through photosynthesis and C release as ecosystem respiration (*R*_eco_) through plant and soil respiration. Whereas much is known about photosynthetic responses to future elevated atmospheric CO_2_ concentrations, and the resulting climatic changes, much still needs to be discovered with respect to the drivers and environmental responses of the respiratory components (e.g., Heinemeyer et al. 2007, 2012). This can be addressed effectively through modeling chamber-based fluxes (Laine et al. 2009). In particular, the temperature sensitivity of soil respiration and its link to canopy activity and C supply is currently intensively debated and researched (e.g., Davidson et al. 2006; Heinemeyer et al. 2007, 2012; Bahn et al. 2008).

Combining automated and multiplexed chamber-based flux approaches with translucent (i.e. Perspex) and opaque chamber types offers a unique opportunity to measure both processes in real time *in situ* and at high frequency. To date, any such combination of translucent and opaque chamber measurements has been performed manually (e.g., Laine et al. 2007, 2009), lacking the necessary high monitoring frequency. Automated systems will ultimately deliver higher temporal flux resolution and thus better parameterization of model process representation, for example, the temperature sensitivity and diurnal changes of respiration and C-flux component contributions. However, chamber artifacts have to be considered, such as lower light levels due to the chamber hood and increases in chamber water vapor and temperatures that will affect photosynthesis, particularly in large chambers (Hooper et al. 2002).

The aims of this study were to (i) field deploy an adapted automated and combined transparent NEE flux and opaque respiration Li-Cor 8100 system in an experimental grass mesocosm study; (ii) use a nutrient fertilization treatment aimed at manipulating NEE fluxes and subsequent mesocosm C balance; (iii) validate the resulting C-balance estimates based on NEE fluxes using measured C stock inventory changes; (iv) assess any chamber artifacts in calculating system C balance; and consequently (v) provide a sound validation for estimating C storage changes using flux chamber approaches. This required the growing of defined vegetation mesocosms on a heterogeneous low C-content soil matrix, monitored using automated translucent and dark chambers, with additional quantification of all C inputs (e.g. as seeds) and outputs (e.g. in drainage water).

## Materials and Methods

### Site description and environmental data

The experiment was performed between 31 October 2006 and 23 January 2007 in northern England at the University of York's experimental garden using scientific sensor equipment (all Delta-T Devices, Cambridge, U.K.). Mean hourly values of temperature (ST1; averaged 30 min. records of 10 min. readings, *n* = 3) at the soil surface, 2 and 5 cm soil depth, soil moisture at 5 cm depth (ML2x; averaged 60 min. records of 10 min. readings, *n* = 1), photosynthetically active radiation (PAR) inside and outside the collar area (one QS sensor each at the central chamber only, averaged 1 min. records of 10 s readings, *n* = 1), wind speed (AN1) and rainfall (RG1) were monitored using a data logger (DL2e) at the site. Air temperature and relative humidity inside each soil chamber were also recorded at each measurement by the Li-Cor flux chamber system (see below).

### Experimental design

**Figure 1:**
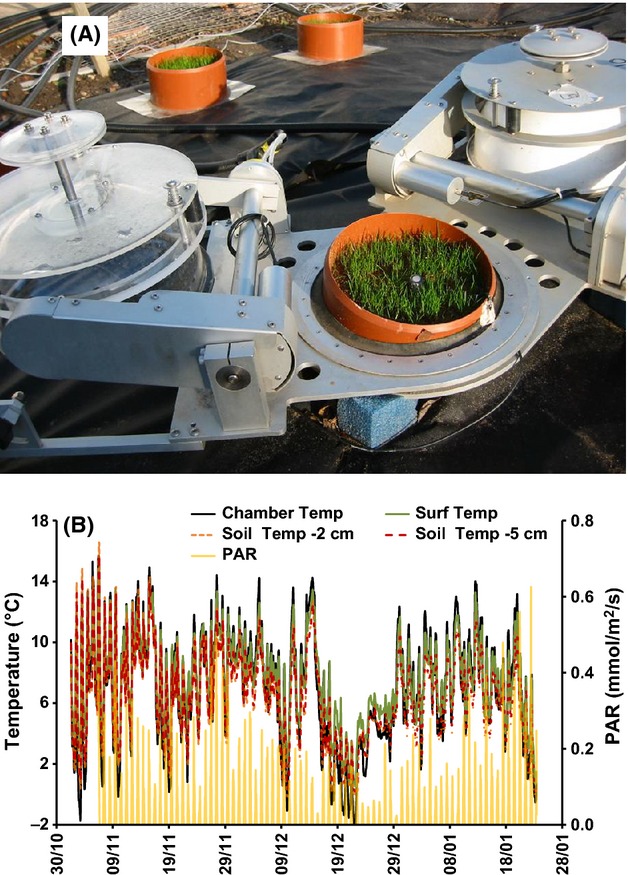
(A) A close-up view of one experimental chamber unit on 8 December 2006, with an experimental mesocosm (with the central light level (i.e., PAR) sensor) monitored by both an opaque and transparent (Perspex) chamber. The black membrane prevented soil splashing and two “harvest collars” are seen in the background. Tilting of the collar limited collar rim shading and improved drainage. (B) Climatic conditions during the experiment showing hourly temperatures measured inside the chamber (Cham Temp), soil surface (Surf Temp) and in 2 and 5 cm soil depth (Soil Temp 2 and 5 cm, respectively) and mean hourly ambient PAR levels (PAR; delayed monitoring start).

An experimental plot within the experimental garden was established during 26–27 October 2006 (see Fig. 1). This plot (5 × 15 m) was divided into three blocks, each containing four plots (each 0.5 × 1.0 m), containing one replicate of a permanent experimental mesocosm combination of either with (+Ch) or without flux chamber (−Ch) and with (+N) or without nutrient (−N) addition (see below). This resulted in four collar treatments: (−Ch −N), (+Ch −N), (−Ch +N), and (+Ch +N), each with three replicates giving a total of 12 (20 cm diameter and 20 cm tall) mesocosms housed inside PVC drain pipe collars (Plumb Centre, Wolseley UK, Ripon, U.K.). The collars had their base glued to a plastic sheet, each with two muslin-covered drainage holes to which a water collection bottle (2 L capacity) with a pressure relief hole was attached via flexible tubing and a connecting T-piece, allowing collection of percolated soil water. The bottle was permanently sunk into the soil, yet allowed access for emptying from one side. The entire plot area was covered with a black weed-suppressing membrane (EAN: 5024160794130, B&Q, U.K.) providing shade and preventing soil splashing from the surrounding area.

Soil was collected on 23 October 2006 from a pine plantation about 10 miles east of York (Allerthorpe Common; 53°91′N, −0°84′W; UK Grid Ref SE752478) on a site previously occupied by lowland heath and selected on the basis of its low SOC content. The soil (Holme Moor series, UK soil classification) is a deep, stoneless gley podzol on eolian sands with a pH_(H2O)_ of 3.5 and a very low C_org_ content (∼0.5%). The soil was taken from the remains of an abandoned badger sett containing plant and root-free B_g_ horizon soil (i.e., largely recalcitrant SOC). The soil was air-dried in a glasshouse, sieved (2 mm) to remove any debris or stones, and the entire volume thoroughly mixed. Each of the 36 collars received a known weight of this soil (*ca*. 2.5 kg). From each collar, 50 g of well-mixed soil sample was taken to determine initial C content (see below). The sandy soil of low C content was chosen to allow detecting mesocosm C stock changes within a short-term experiment; reliably detecting soil C changes in higher C_org_ soils becomes methodological near impossible due to soils' inherent spatial heterogeneity. Such detection limits would only hinder the experimental testing of the hypothesis, and the presented setup is to be seen as an experimental platform to validate detecting measurable C stock changes under field conditions.

Seeds (*Lolium perenne* L.; B&Q, U.K.) were sown on 3 November 2006 at a rate of *ca*. 3.1 g DW per collar area (278 cm^2^) corresponding to 112 g DW m^−2^. After sowing, seeds were covered with a fleece to prevent rain splash and bird predation until germination. In order to determine the C entering the cores from the seeds, six ∼3.5 g DW seed aliquots were taken for total C analysis (see below).

Half the experimental mesocosms received four applications of Hoagland's nutrient solution (20 mL each) from a full strength volume (1 L) kept at ambient temperature during the experiment (8, 16 and 21 December 2006 and 8 January 2007, respectively). The other half received similarly 20 mL of deionized water at the same temperature.

### Soil and net ecosystem CO_2_ flux measurements

We used a closed dynamic soil CO_2_ flux system (Li–Cor 8100, Li–Cor, Lincoln, NE) for measuring CO_2_ flux rates (*μ*mol CO_2_ m^−2^ s^−1^) on the experimental +Ch plots. We monitored a short period (31 October until 3 November) before grass seeds were added (i.e., soil respiration only) using dark chambers, and flux rates were calculated as the linear CO_2_ increase (1 s readings) during closure time (135 s), discarding at least a 20 s initial “dead band” mixing period (see Li-Cor 8100 manual). The automated system allowed 12 long-term chambers (model: 8100-101; 20 cm diameter) to be linked to the Li-Cor 8100 infrared gas analyzer unit via a custom-built multiplexed gas handler unit (Electronics Workshop, Biology Department, University of York, U.K.), allowing hourly measurement cycles with 1 min 25 s sampling delay between chambers within a 20 m diameter (see Heinemeyer et al. 2007).

After seed addition (3 November) only combined soil and plant fluxes were monitored. In order to measure NEE fluxes, half the Li-Cor long-term chambers were adapted by replacing the chamber lid and most mechanical parts with (4 mm thick) equivalent parts in Perspex® (Cast Perspex®; York Plastics, York, U.K.); the other half was left dark. This allowed breaking down the NEE flux into ecosystem C uptake and C release (*R*_eco_ in the dark during day and night) and exploring different responses to temperature and developmental effects over time. Two chamber base rims (of one dark and one transparent chamber) overlapped, and the chambers being placed around the collar at an angle of 110° to limit shading of the collar area by the chambers. Additionally, collars (but not the soil level) were slightly tilted (∼5 degree to the true plane) to allow better drainage and limit collar and chamber shading of the soil area (see Fig. 1). In any measurement cycle, two chambers, first the transparent and then the dark chamber, were monitored in turn at each experimental collar; monitoring then switched to the next collar, enabling for CO_2_ flux to be measured for all six experimental collars on an hourly cycle. Flux calculations were performed routinely using the Li-Cor 8100 software (version 1.3.0), with volumes adjusted to include multiplexer and tube air volumes and individual collar offsets (i.e., rim height above soil surface). The system thus allowed both NEE fluxes and its respiration component to be measured continuously, resulting in either positive (net CO_2_ release) or negative (net CO_2_ uptake) fluxes. Calculation periods were 90 s, with a starting period of 50 s for dark (reflecting continuation of photosynthesis during initial chamber closure) *vs*. 20 s for NEE (reflecting immediate photosynthesis) chambers. Throughout the manuscript the micrometeorological sign convention for NEE is used (unless otherwise stated), in which a net flux from the biosphere to the atmosphere is positive, also corresponding to the Li-Cor software calculations.

### Shoot harvesting

All shoots of the replicates from each of the +N and −N mesocosms were cut off at soil level on 23 January (final harvest). Leaf area (LA) was measured by scanning at 150 dpi using an Epson Perfection 4870 scanner and LA then analyzed in WinRhizo® 4.1c (Regent Instruments Inc., Quebec city, Quebec, Canada). Subsequently, leaf fresh weight and dry weight (after oven-drying for 3 days at 65°C to constant weight) were recorded, and samples retained for total C analyses (see below).

### Root and soil harvesting

All roots from the replicates from each of the +N and −N mesocosms were extracted on 23 January 2006 (final harvest). PVC mesocosms were taken from the field site to the laboratory; roots were extracted (using tweezers) on plastic trays and the soil then washed on a 710-*μ*m mesh; remaining roots being extracted and dried using a paper towel. Total fresh weight of extracted roots (taking extreme care to ensure removal of any remaining soil) was measured; dry weight was then determined (as above). A subsample of 50 g DW of the remaining soil was taken from the well-mixed total soil volume (before washing for remaining root extraction) and kept in air-tight jars after oven-drying as previously described.

### Carbon analyses and budget calculations

Seeds and husks plus roots each were first milled in a ball mill to a fine powder before being analyzed at the University of York, Biology Department, using an EA FlashEA1112 (Thermo Finnigan, Bremen, Germany) unit, linked to a custom-built IR-MS (a standard laboratory gas chromatograph is coupled to a 12 cm radius magnetic sector mass spectrometer (SIRAS Series2, Micromass, U.K.), nonionizing electromagnetic radiation (NIER) type ion impact source, triple faraday collector system, rotary/turbo-molecular pumping vacuum system; constructed by Pro-Vac Services Ltd., Crewe, U.K.). Soil and shoots were also milled and analyzed using an elemental C/N analyzer (Shoots: Carlo Erba NA 2500; Perkin Elmer, Cambridge, U.K.; Soil: Vario Macro, Elementar, Hanau, Germany) at Edinburgh and York University. Total dry weight of the analyzed material was about 3.5 mg for seeds and husks, shoots and roots, and 100 mg for soil. The C-content variability (based on standard deviation of replication of standards) was 0.005% C_org_ (equal to 0.15 g C detection limit per mesocosm).

Dissolved organic carbon (DOC) analysis was performed on the bottle-collected water samples (which were kept in a refrigerator until analysis the same week) using a Liqui TOC II (Elementar) analyzer at the University of York Environment Department with a detection limit (based on reference standards) of <1 mg L^−1^.

We calculated the C balance of the flux measurement approach as the cumulative sum of hourly fluxes during the entire experimental period, including the mesocosm's C_DOC_ flux. For the C stock inventory calculation, we summarized the individual mesocosm C pools (note: the negative multiplier allows direct comparison with the C-flux estimate):


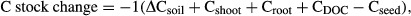
(1)

where ΔC_soil_ is the differences between initial and final soil C content, C_shoot_, C_root_, C_DOC_, and C_seed_ are the C content in shoot, root, DOC, and seeds, respectively.

### Statistical analysis

Statistical analyses were carried out using SPSS (version 18; SPSS Science, Birmingham, U.K.) with Kolmogorov–Smirnov and Levene's tests being used to check for normality and homogeneity of variances. Individual one-way ANOVAs were carried out for the cumulative sums of NEE over the entire experimental period in order to test for differences in the C balance between treatments. As there were no significant differences between either nutrient or chamber treatments for any parameter, the replicates were pooled (i.e., providing *n* = 6). Significant differences in the shoot and root biomass, C-content data and final C-balance estimates based on NEE fluxes versus C stocks were based on one-way ANOVA. To calculate and detect differences between temperature responses of respiration fluxes (i.e., Q_10_s), we followed the methods outlined in Heinemeyer et al. (2012).

## Results

### Collar establishment

The soil membrane (Fig. 1A) successfully prevented any soil (and thus C) splashing into the collar areas and the fleece protected against seed loss through bird predation or rain splashing. Weather during the experimental period was relatively cold and wet, with snow on the last day of the experiment; this led to relatively slow plant growth. Percolated water in the large bottles allowed collection of accumulated rainfall after rainy periods on three occasions: 4 (∼650 mL) and 19 (∼850 mL) December 2006 and 23 (∼1700 mL) January 2007. One issue was that the low sun angle during the period of the experiment led to overall low light levels and a tree shadow moving over the plot area.

### NEE chamber performance

**Figure 2:**
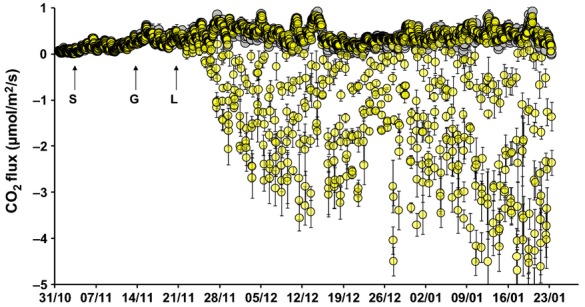
Mean CO_2_ fluxes ±SE (*n* = 6; ignoring the nonsignificant N treatment) from the opaque respiration (gray circle) and transparent NEE chambers (yellow circle) during the entire experimental period. The timings of sowing (S), germination (G), and first leaf stage (L) are indicated by arrows.

The dynamic chamber system performed reliably throughout, providing continuous hourly and NEE and *R*_eco_ flux measurements (Fig. 2). However, the transparent chambers were cleaned at least weekly to prevent buildup of dust and dirt. The temperature increase inside the transparent chamber during daylight chamber closure was limited during this winter period to less than 2°C; it nonetheless also reduced relative humidity levels by about 5–10% over the 90 s flux period at midday (data not shown).

The CO_2_ flux data showed clear differences in the time course for the opaque versus transparent flux chambers and consequently the chosen time periods used for the flux calculations. Firstly, the dark chamber fluxes needed a longer “dead band”, apparently due to photosynthesis continuing for a short period even after chamber closure during peak light periods (although line flushing time and air mixing could also cause such a delay). Secondly, transparent chambers showed a near instantaneous decline in CO_2_ concentration during light (albeit a short increase could frequently be observed about 20 s after chamber closure), which sometimes was reduced over time (possibly due to CO_2_ draw down); they thus required a shorter “dead band”, only reflecting chamber air mixing. However, both chamber fluxes were calculated as a linear CO_2_ change over the subsequent 90 s.

**Figure 3:**
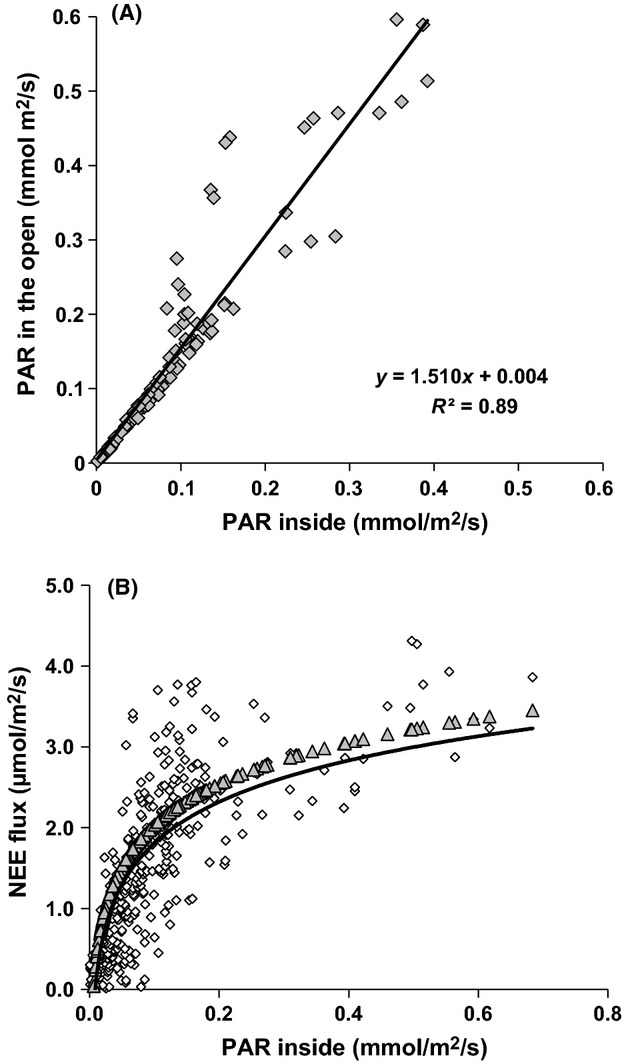
(A) Comparison of PAR levels outside versus inside the monitored transparent chamber (during 6 – 29 November 2006) used to calculate net ecosystem exchange (NEE) chamber light level (i.e., PAR) underestimation. The best-fit regression (linear) function (*P* < 0.001) and corresponding adjusted *R*^2^ are shown. (B) Correction of negative NEE fluxes (note: negative C-uptake fluxes shown as positives because of the regression) inside the PAR monitored chamber is based on the calculated PAR reduction (∼34%) and the relationship between NEE versus PAR. The white diamonds indicate 340 measured NEE fluxes during December 2006 till January 2007 (filtered, e.g., excluding nighttime and rainy periods). Also indicated are the resulting correction offset (gray triangles) of adjusted NEE fluxes versus previous uncorrected regression values (black regression line; *y* = 0.74 Ln(x) + 3.51, *R*^2^ = 0.59).

PAR reduction caused by the Perspex lid as measured at the central plot was considerable over a wide range of PAR levels; overall, a PAR reduction in ∼34% was observed (Fig. 3A); the nominal transmission for this Perspex 3 mm is 90% for visible light, but thickness and light angle reduce this and we measured PAR. Additionally, the noise in Fig. 3A is thought to result from patchy and moving shadow effects (e.g., from higher tree branches) or temporary presence of dirt on the chamber. Fig. 3B shows a plot of NEE flux against PAR (note that negative C-uptake NEE fluxes are shown as positives for regression purposes), allowing calculation of a light response curve regression (see equation in legend to Fig. 3B) that provided the basis for estimating the impact of reduced PAR beneath the Perspex domes on NEE in fluxes. This information was used to correct observed daytime transparent chamber NEE fluxes during the entire period of net C uptake (i.e., 21st November 2006 onwards), based on the NEE offset between the two regressions (uncorrected *vs*. corrected regression) over the entire positive (i.e., light) PAR range (Fig. 3B). Although the individual NEE flux corrections were mostly small (applied to light-period NEE fluxes from 25 November onward), the overall effect on the total cumulative NEE flux was considerable. Uncorrected *vs*. corrected cumulative mean hourly NEE fluxes (± SE; *n* = 6) over the experimental period was −55.0 ± 10.6 versus −152.0 ± 10.6 *μ*mol m^−2^ s^−1^, respectively.

### Plant growth

Plant establishment was slow, with subsequent growth being limited by cold weather (Fig. 1B). Seedlings germinated on 14 November, giving rise to a 1-cm-long brown stalk by 17 November; first shoots were recorded on 20 November and were 2 cm long 1 week later. Consistent increases in leaf area (LA) and in shoot and root dry weights (SDW, RDW) were observed throughout the experiment (determined from additional nonmonitored sequential “harvest collars”; see Fig. 1A, data not shown); nutrient treatment did not have any significant impact on any growth parameter at any harvest. At the final harvest, 81 days after planting (31 October 2006), the combined LA was 556 cm^2^, SDW was 1.75 g, and RDW was 2.43 g.

### Carbon content analyses

Soil, seeds, and husks plus roots had C_org_ contents of around 0.5, 41.5, and 34.5%, respectively. Shoots had a C_org_ content of *ca*. 40.5%, and DOC samples showed a C_org_ concentration of *ca*. 20 mg L^−1^. However, calculated total soil C_org_ content changes (excluding root C of ∼0.8 g) of about 0.015 g C (see Table 1) during the period in this very low C soil were small and within the analytical detection limit of 0.15 g C per mesocosm soil volume (see Carbon analyses and budget calculations).

**Table 1:** Comparison of final C balances in g C per mesocosm ± 1 standard deviation using the inventory (Stock = −1(ΔC_soil_ + C_shoot_ + C_root_ + C_DOC_ − C_seed_) and flux approach (Flux; fluxes are PAR corrected (see Fig. 3) and include C_DOC_) across the different treatments, that is, ± chamber (±Ch), and in combination with ± nutrient (N) addition (±Ch±N).

Treatment	(*n*)	C_soil_	C_shoot_	C_root_	C_DOC_	C_seed_	Stock C balance	Flux C balance
−Ch	6	0.021 ± 0.142	0.706 ± 0.017	0.803 ± 0.039	0.054 ± 0.002	1.299 ± 0.001	−0.286 ± 0.158	n.a.
+Ch	6	0.012 ± 0.063	0.716 ± 0.037	0.821 ± 0.076	0.055 ± 0.003	1.298 ± 0.001	−0.305 ± 0.066	−0.305 ± 0.042
+Ch −N	3	0.040 ± 0.055	0.737 ± 0.012	0.786 ± 0.049	0.054 ± 0.004	1.298 ± 0.001	−0.320 ± 0.088	−0.310 ± 0.052
+Ch +N	3	−0.017 ± 0.067	0.694 ± 0.043	0.856 ± 0.092	0.056 ± 0.002	1.299 ± 0.002	−0.290 ± 0.050	−0.300 ± 0.040

Individual treatments (i.e., ±N) are also shown as combined values (i.e., +Ch) as there were no significant differences for any chamber or nutrient treatment combinations; n.a. denotes not measured.

### Respiration chamber fluxes in the dark and temperature sensitivity

**Figure 4:**
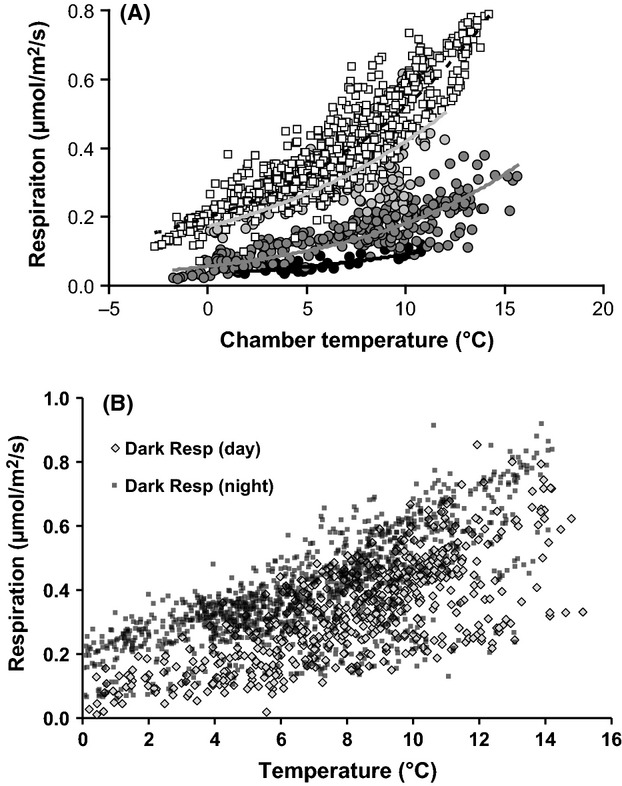
(A) Opaque chamber dark period respiration fluxes versus chamber temperature during the entire experimental period after germination. Data are divided into four stages: presowing/soil only (S1) (31 October – 3 November 2006; black circles; Q_10_ = 2.30 ± 1.12 SE), germination (S2) (4 November – 13 November 2006; dark gray circles; Q_10_ = 3.22 ± 1.04), seedling (S3) (14–27 November 2006; light gray circles; Q_10_ = 2.45 ± 1.05), and plant stage (S4) (28 November 2006 – 23 January 2007; open squares; Q_10_ = 2.65 ± 1.02). Each best-fit exponential regression line (symbols corresponding to line colors, all regressions *P* < 0.001) reflects unique temperature (this was chamber temperature for all, but the S1 stage which was soil temperature at 5 cm depth) sensitivity (Q_10_) with S1: *y* = 0.04 e^0.10x^, *R*^2^ = 0.79; S2: *y* = 0.06 e^0.12x^, *R*^2^ = 0.72; S3: *y* = 0.17 e^0.09x^, *R*^2^ = 0.59; S4: *y* = 0.19 e^0.10x^, *R*^2^ = 0.82. (B) Respiration fluxes from opaque chambers (Dark Resp) versus chamber temperature during the experimental period after seed germination (3 November), separately for nighttime (night) and daytime (day) fluxes.

Based on using only the opaque chamber fluxes, we calculated the apparent temperature sensitivity of ecosystem respiration fluxes (i.e., increase in flux over a 10°C rise; Q_10_). Measured fluxes during the preseed period (i.e., only soil) correlated better with soil temperature at 5 cm depth, whereas after sowing chamber air temperatures correlated best with measured fluxes (Fig. 4A). Four distinct periods were observed with different Q_10_s (see Fig. 4A), the presowing (i.e., soil only) stage (Q_10_ = 2.71) having a lower sensitivity than the germination stage (Q_10_ = 3.22), which showed the highest sensitivity. In addition, once plants were fully established (Q_10_ = 2.65) another slight increase was observed compared with the seedling period (Q_10_ = 2.45), which showed the lowest sensitivity. However, the observed differences in Q_10_s were not significantly different (see ±SE in legend to Fig. 4A). Moreover, an overall comparison of ecosystem respiration fluxes in the dark revealed reduced respiration rates during the day compared with nighttime respiration fluxes at the same temperatures (Fig. 4B).

### Transparent chamber fluxes and light response

Although the transparent chamber fluxes had to be corrected for reduced light levels at the time of NEE fluxes, they provided a high temporal resolution dataset on the light response, indicating that during this study maximum photosynthesis rates seemed to be reached at PAR levels of just above 800 *μ*mol m^−2^ s^−1^, which was indicated by NEE asymptotically reaching near saturation levels at this range (Fig. 3B). Further, the PAR compensation point of NEE based on the regression in Fig. 3B was just under 10 *μ*mol m^−2^ s^−1^, although relatively low, this is similar to data reported by Nijs and Impens (1996) for *Lolium perenne*, particularly considering the low temperatures reducing respiration rates together with a lack in nutrient limitation (see Assessing the fertilization effect) supporting efficient, temperature acclimated net photosynthesis rates in this experiment.

### Assessing the fertilization effect

**Figure 5:**
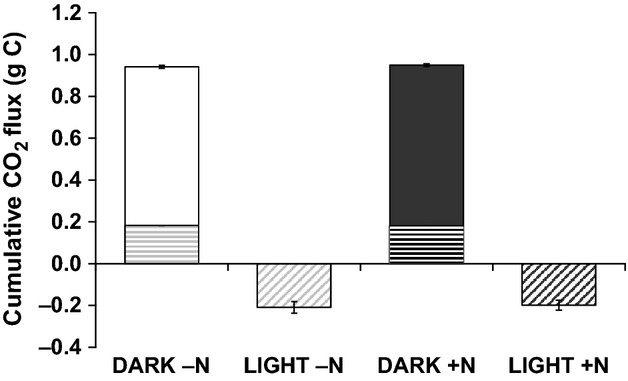
Cumulative hourly PAR uncorrected chamber CO_2_ fluxes ± STDEV (*n* = 3) over the entire experimental period 3 November 2006 – 23 January 2007 for opaque (Dark) (including the estimated heterotrophic flux component (horizontally striped area) based on the Q_10_ relationships, see Fig. 5) and transparent (Light) chambers; ± N indicates nutrient fertilization treatment, which did not show any significant effect.

Plants did not show any visible nutrient fertilization effects, which was confirmed by the plant growth parameter comparison (Table 1). Moreover, neither the cumulative hourly net C fluxes (Fig. 5) nor the stock inventory based C balance (Table 1) showed any significant fertilization effect.

### Estimates of the total C balance: in situ NEE vs. C stock approach

**Figure 6:**
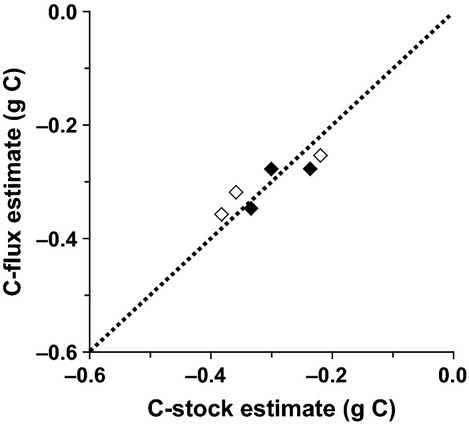
C balance of the individual chamber NEE fluxes (from the individual mesocosm collar locations; fluxes are PAR corrected, see Fig. 3 and including C_DOC_, see Table 1) compared with their corresponding C stock inventories. Open and closed symbols correspond to −N and +N nutrient treatments, respectively. Also shown is the 1:1 line.

There was no significant difference in the final C stock of experimental mesocosms with or without chamber presence (Table 1, comparing +Ch vs. −Ch stock inventories); thus, flux monitoring did not interfere with the total C fluxes. Importantly, comparison of flux-based (including PAR correction and C_DOC_) versus stock-based C balances did not show any significant differences either (Table 1, comparing Flux ±N vs. Stock all). However, this included very small average changes of about 0.015 g C in total mesocosm soil C of ∼12.50 g C (∼2500 g sand with a C_org_ content of ∼0.5%), which was within the analytical detection limit of 0.15 g C (see Table 1 and sections Carbon analyses and budget calculations and Detecting changes in the C balance). DOC contributed only a small amount (0.05 g C) to the net C budget of −0.31 g C, and root and shoots contributed 0.81 and 0.71 g C, respectively, compared with the initial seed C of 1.30 g C. Overall, the comparison of flux-based *vs*. stock-based C-balance estimates per experimental mesocosm (Fig. 6), including the nutrient treatment mesocosms (*n* = 6), showed good agreement with the 1:1 line, notwithstanding the relatively small C-balance range.

## Discussion

### Chamber performance

Overall, the combined flux system performed extremely reliably, without any chamber failures (Fig. 2), enabling an uninterrupted estimate of hourly C exchange using a flux-based approach. Importantly, the presence of the chambers did not affect the C balance of the experimental systems (Table 1). However, chamber-based monitoring requires careful consideration of several issues, including chamber positioning and avoidance of collar or chamber shading effects. For example, the larger error bars seen in the daytime transparent NEE fluxes presented here result from partial shading across the plot, affecting photosynthesis. The temperature increase, and associated reduction in humidity, inside the transparent, compared with the opaque, chambers was not an important issue in this study with very short closure times (∼2 min), although it has been noted as a potential problem (Hooper et al. 2002); clearly, this would become more important at longer closure times. Under warmer conditions (season or climatic region), a temperature control might need to be fitted to the Perspex system (see Laine et al. 2007, 2009). In our study, neither plant growth nor C balance (Table 1) was significantly affected.

An important consideration was the selection of the time interval used for flux calculations; this differed between opaque and transparent chambers and required consideration of the chamber air mixing and photosynthetic lag periods. However, even more important was the need to correct for the considerable PAR reduction, of around 34% (Fig. 3A), resulting from attenuation through the Perspex chamber, resulting in corrected higher net C uptake (Fig. 3B). The nominal transmission reduction was reduced further due to thickness and most likely the light angle (less effect at higher sun angles); although thinner Perspex might improve this, it will compromise strength. Importantly, actual *in situ* PAR transmission should always be monitored to allow correcting for “real” conditions. The positive finding here is that appropriate corrections can be derived and applied to NEE data, resulting in C-flux data that match almost exactly the independent data gained from measuring C stock inventories. Overall, the PAR correction resulted in a 2.8-fold increase in the C balance, which seems very large, reflecting the duration of the experiment over which the individual flux corrections added up. However, we acknowledge several potential shortfalls in our NEE flux correction calculation, adding to the overall flux uncertainty rather than questioning the validity of this study. Chamber PAR corrections are frequently made with NEE chambers based on such asymptotic PAR relations of NEE (e.g., Risch and Frank 2006) as PAR can be seen as the main determinant of NEE (Burrows et al. 2005), autocorrelating with temperature. Indeed, our plot of NEE response to PAR (*R*^2^ of 0.6) is very similar to a reported study (see Fig. 5 in Burrows et al. 2005), and such an overall PAR adjustment has been suggested previously (Vogel et al. 2009). Further, the fit of NEE versus PAR could be performed by different models, which could impact on the corrections (a hyperbola fit did not result in a better model). Moreover, collar to collar variation was not accounted for (we only had one set of PAR sensors for one centrally located chamber) but was deemed to be negligible as all chambers were newly manufactured from one mold and checked daily for any dirt accumulation and cleaned regularly.

### Combining dark and transparent chambers for measuring flux components and their environmental responses

The high frequency of dark chamber flux measurements allowed calculation of periodic apparent Q_10_ values for *R*_eco_, showing trends of changes in the system's Q_10_ reflecting different sensitivities of developmental stages (e.g. germination). Seed germination and establishment are known to be very sensitive to temperature (Shen et al. 2008). The preseed period allowed the heterotrophic soil respiration component, and its temperature sensitivity, to be determined before any autotrophic C inputs to the mesocosms. We did not continue monitoring soil heterotrophic respiration, which might have been altered through soil priming via root C inputs; however, there is currently no perfect method available to separate soil C-flux components (Heinemeyer et al. 2012). There was a clear increase in overall respiration and in its temperature sensitivity after seed germination (albeit not significant, see ±SE of Q_10_s in Fig. 4A), reflecting the increased metabolic activity and available seed starch energy reserves. This change occurred abruptly (i.e., shift in respiration rates), reflecting the bulk seed germination. This analysis should not be seen as a valuable temperature responses analysis *per se*, but rather shows the opportunities of exploring this chamber setup to gain improved process-level understanding within *in situ* ecological studies of established systems. Moreover, this setup also allows an important comparison of *R*_eco_ in the dark during the day versus night, and our data confirm a strong respiration inhibition during the grass growth period (e.g., Atkin et al. 1998) during the day (Fig. 4B). We did measure respiration in the dark shortly after chamber closure (darkening over ∼2 min due to slow chamber closure), thus any postillumination burst effects should have been prevented (i.e., Atkin et al. 1998). Although we noticed such a short-term increase at about 20 s after chamber closure, this was outside the flux calculation period and is likely only important for physiological leaf-level studies and does not have any implications on the calculated NEE fluxes.

### Detecting changes in the C balance

We selected the soil type in order to have low (less than 1%) background and mainly older, recalcitrant SOC content with no dead root presence causing inherently spatially variable C_org_ content; the calculated changes in SOC content were very small and, although within the detection limit (C_org_ ±0.005%), indicated no significant change in SOC. The precision of determining total mesocosm SOC was 0.15 g C, about half of the derived entire mesocosm C-balance change. However, the use of even more accurate C analysis instruments will do little to overcome a generally high spatial variability in SOC (Niklaus et al. 2000), and our well-mixed and low SOC system seems to have assured the needed accuracy to compare both approaches *in situ*. An increase in SOC due to root litter over this short-term study was unlikely as root growth of the slow-growing grass was limited, and no dead roots were observed during extraction, and any exudates would have mostly been leached out from the sand into and captured by the DOC collection bottles. Notwithstanding any possibly undetected small SOC changes, this issue highlights the difficulties in determining short-term changes in C stock-based inventories, due to the inherently high variability in SOC content and, thus, the need for a reliable flux-based C-balance approach (Niklaus et al. 2000; Rodeghiero et al. 2009). Moreover, although overall DOC fluxes (only about 0.02% of the total flux balance) were a small part of the C balance, as proposed by Niklaus et al. (2000), they improved the comparability of the two approaches in our study and could be important in established ecosystems. However, we acknowledge that we did not measure potential C losses as VOCs or methane, the former can be assumed negligible as no mowing took place, and the latter was unlikely as we did not work in waterlogged soils. Nevertheless, in peat systems, methane emissions could be accounted for by linking this flux system to suitable analyzers via subsampling the air stream (e.g., Los Gatos Inc., Picarro Inc.)

Overall, plant growth showed a net C gain through the active growth period (data not shown) with more light and higher temperatures in November (Fig. 1B), but visually slowed down by the end of the experimental period, likely due to colder and less bright weather conditions. Neither the net C-flux- (Fig. 5) nor the C stock-based carbon balance (Table 1) showed any significant nutrient fertilization effect. However, this could be explained by low plant growth rates under the relatively cold conditions and consequent adequate nutrient supply which still resulted in six overall data points for C-balance comparison. The large seeds of *L. perenne* will also have provided essential nutrients to support the early stages of growth.

### Estimates of the total C balance: in situ NEE vs. C stock approach

The lack of any significant difference in the final C balance of the mesocosms with or without chamber presence (Table 1, comparing +Ch vs. −Ch stocks) indicated that measurements did not interfere with the C fluxes, after appropriate allowance had been made for PAR reductions. To our knowledge, such a test has never previously been performed. Importantly, the overall comparison of the flux-based *vs*. the stock-based C balance (Table 1, comparing +Ch stock vs. flux), or the individual collar-based comparison of flux-based *vs*. stock-based C-balance estimate (Fig. 6), did not show any significant differences.

### Implications for field measurements of NEE fluxes and modeling

A major advantage of this combined dark and transparent flux chamber system is disentangling the net C fluxes and their unique environmental responses, a particular challenge in modeling environmental responses of ecosystem C fluxes (Davidson et al. 2006). It can provide crucial insight into model parameters, such as Q_10_ values or light response curve parameters, that (in long-term studies) could reveal potentially important developmental and seasonal effects. Insights into developmental, phenological, or seasonal stages of C-uptake *vs*. C-release processes would then lead to improved models with greater predictive power; our short-term Q_10_ analysis should only be seen as an example for such analysis. Of particular interest is the impact of plant C uptake and its allocation to the rhizosphere, leading to resulting changes in decomposition (i.e., priming) (Fontaine et al. 2003) and resulting apparent versus intrinsic temperature sensitivity of measured soil C efflux (Wang et al. 2010). Further attention should also be paid to considering model structures when collecting field chamber fluxes as most soil carbon models only predict a decomposition soil C efflux (Heinemeyer et al. 2010); as such additional root-free plots for dark chamber decomposition fluxes would add valuable data to model validation and development.

### Limitations, further improvements, and application ideas

Although our opaque PVC collars were tilted, they still caused some limited shading inside during low sun angle; clearly, it would be better to use Perspex collars to reduce further the collar shading effects, particularly at low sun angles. Another collar issue for consideration under natural conditions is the avoidance of cutting roots on collar insertion, because this potentially reduces considerably root-derived fluxes (Heinemeyer et al. 2011). Moreover, changes in chamber temperature and humidity during high transpiration rates can affect stomatal conduction and thus photosynthesis (Hooper et al. 2002), although in our cold season study, this was less of a problem, this might need to be addressed under different environmental or ecosystem conditions. All these factors are also affected by chamber size; a larger system may require mixing fans to ensure adequate CO_2_ supply across the leaf boundary layer, with inherent concerns about pressure impacts and concomitant abnormal soil CO_2_ efflux (Lund et al. 1999). Our system ensured adequate air mixing by an integral air pump inside the CO_2_ analyzer that maintained adequate linear uptake in photosynthesis while minimizing disturbing the soil diffusion gradient.

The combination of dark and transparent chambers also allows GPP (NEE – *R*_eco_) to be determined (not shown here), which normally relies on regression model predictions (e.g., CarboEurope IP: http://gaia.agraria.unitus.it/database/eddyproc/). These data enable *in situ* comparison with model predictions that are based mainly on (to daytime) extrapolated nighttime respiration temperature regression models, at least in low vegetation systems. Moreover, the combined chamber system also revealed the often suggested but yet poorly documented and understood the phenomenon of suppressed leaf respiration in the dark during the day (Fig. 4B); therefore, such data could provide better estimates of GPP, that is, without the need for extrapolating nighttime respiration data to daytime. A further use of the automated transparent chamber fluxes is the high temporal resolution available for obtaining *in situ* light response curves for a well-defined vegetation type, suitable for upscaling fluxes to the landscape scale or assessing spatial variability within eddy covariance C-balance footprints (Fox et al. 2008), albeit so far only applicable to short vegetation. Certainly, a multiplexed chamber system, as presented here, can provide valuable insights into small-scale variability in the net C-balance flux components across low vegetation ecosystems (e.g., Subke et al. 2012), at a scale not captured by larger C footprint systems such as eddy covariance (Fox et al. 2008).

A further added advantage of our system is the possibility to monitor water fluxes (which are needed for CO_2_ dry calculations, see Li-Cor 8100 manual), allowing high frequency *in situ* field measurements of actual evapotranspiration (AET) to be obtained as reported by Stocker et al. (1997). Importantly, water fluxes are known to relate to C turnover, with demonstrated correlations between AET and NPP (Webb et al. 1978), litter decomposition (e.g., Minderman 1968; Meentemeyer 1978), and thus C-cycle models (e.g., Heinemeyer et al. 2010). However, obtaining accurate measurements of AET is difficult, although this has been attempted using eddy covariance systems (Gielen et al. 2010) and is particularly appropriate when using an automated dynamic closed chamber flux approach (over low vegetation). Such AET data could usefully be compared with eddy covariance water fluxes (i.e., latent heat flux), thus helping to address the associated large uncertainty in these water balance estimates (Gielen et al. 2010). They could also be combined with *in situ* chamber-based litter decomposition studies, capturing both C and water fluxes.

## Conclusions

Summarizing, our evaluation of C-balance estimates from an automated high frequency ground-level flux chamber system showed:

1. Automated NEE flux chambers provided accurate C-balance estimates compared with C stock inventories in short grass mesocosms. Moreover, chamber presence did not affect plant growth or alter the mesocosms' net C balance.
2. A combination of transparent and opaque chambers obtained reliable ecosystem NEE data and its respiration components, suitable for model validation.
3. Whereas transparent chambers provided *in situ* light response curves, opaque chamber fluxes allowed estimation of individual growth stage ecosystem Q_10_ values and detecting suppression of leaf respiration during the day, both crucial for future C-flux model developments at high temporal resolution.
4. It is imperative to correct for light-level reductions in NEE chambers, and other changes in the chambers' environmental conditions need to be addressed appropriately for other chamber sizes or environmental conditions (e.g., cooling in hot environments, air mixing in larger chambers).
5. Such a combined chamber system has a particular potential to enhance our understanding of C-cycling processes in low vegetation and potentially for water flux monitoring, as well as to address eddy covariance footprint issues and assumptions used for their respective GPP calculations.

Although the overall combined chamber concept and its applications at process level should hold true in general, it will require careful consideration in the field to address ecosystem and site-specific demands.
